# Supplementary material for: Adaptive behavior can produce maladaptive anxiety due to individual differences in experience
Source: Evol Med Public Health. 2016 Aug 16;2016(1):270–85. doi: 10.1093/emph/eow024 (PMC5490257; doi:10.1093/emph/eow024)
Supplement: Supplementary Data [file supp_2016_1_270__index.html]

Adaptive behavior can produce maladaptive anxiety due to individual differences in experience — Supplementary Data 

# Adaptive behavior can produce maladaptive anxiety due to individual differences in experience

## Supplementary Data

files

- Supplementary Data - zip file
