# Supplementary figures and images for: Adaptive behavior can produce maladaptive anxiety due to individual differences in experience
Source: Evol Med Public Health. 2016 Aug 16;2016(1):270–85. doi: 10.1093/emph/eow024 (PMC5490257; doi:10.1093/emph/eow024)

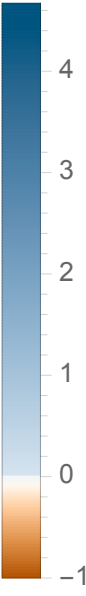

Supplement: Supplementary Data [file supp_eow024_suppl_data.zip › SensitivityToPgPbRatioScaleBar.pdf]

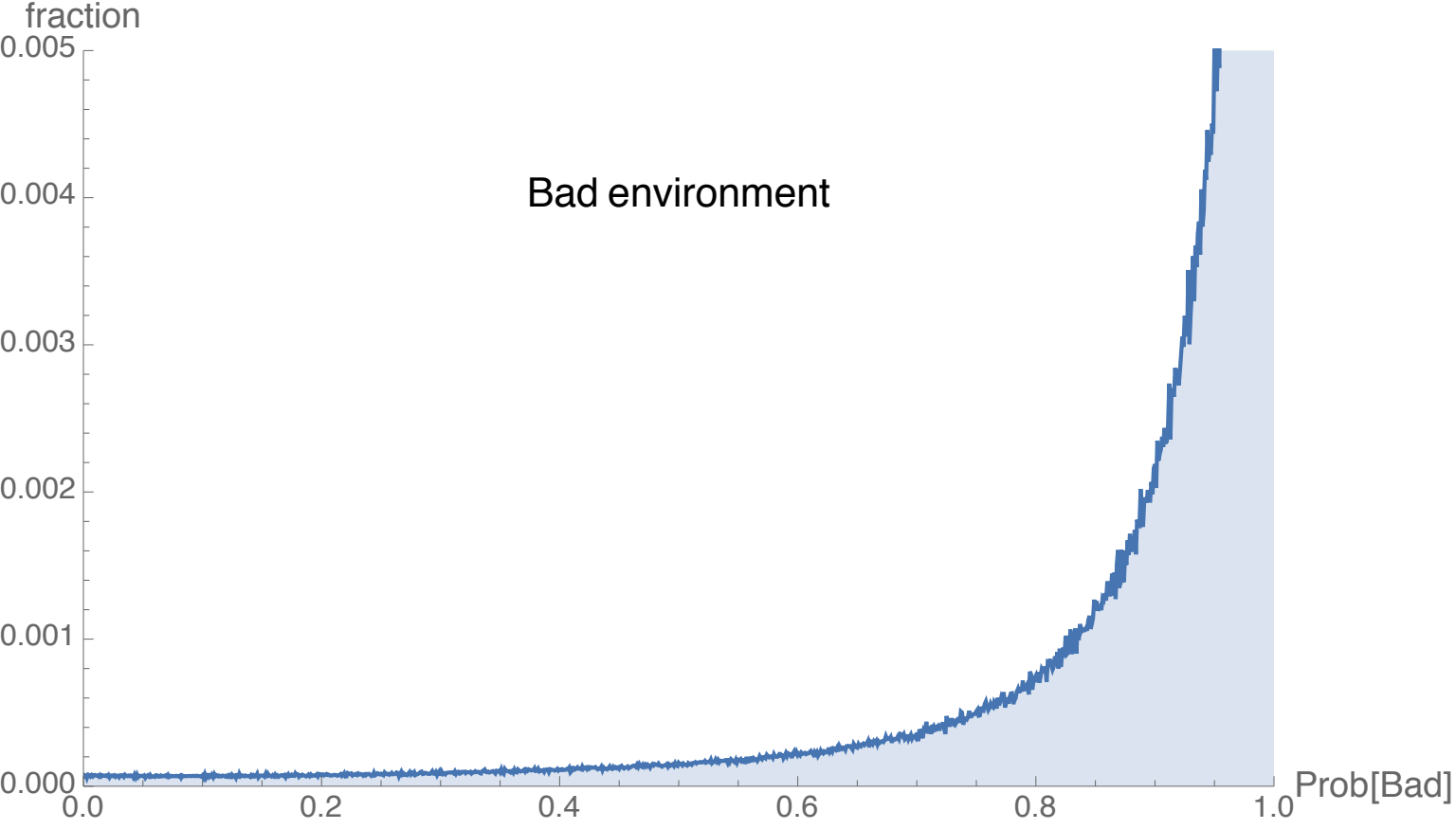

Supplement: Supplementary Data [file supp_eow024_suppl_data.zip › BadEnvironment.pdf]

# Bad Environment

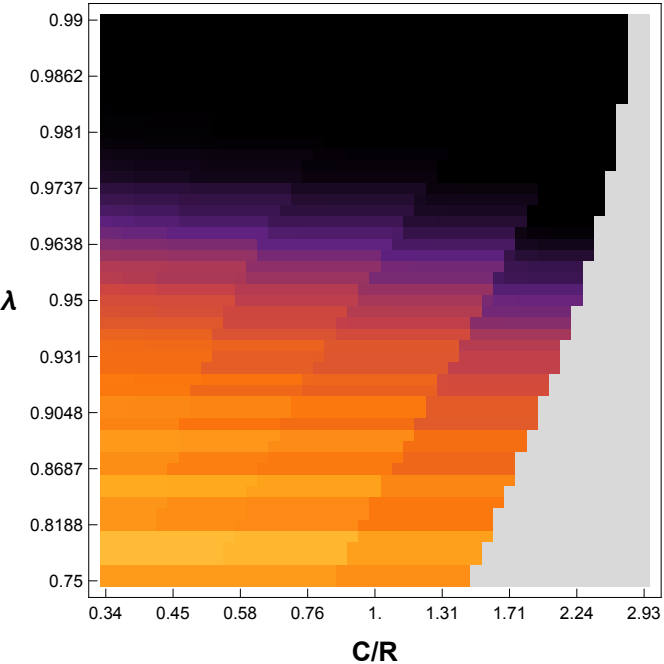

# Good Environment

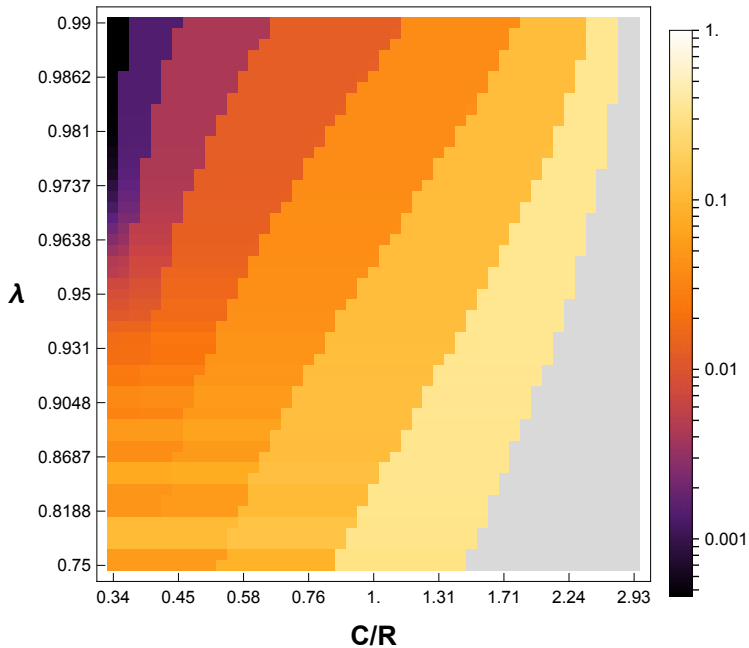

Supplement: Supplementary Data [file supp_eow024_suppl_data.zip › DCBadGoodFigureLabeled.pdf]

Log ratio of fraction mistaken in Good:Bad

$\lambda$

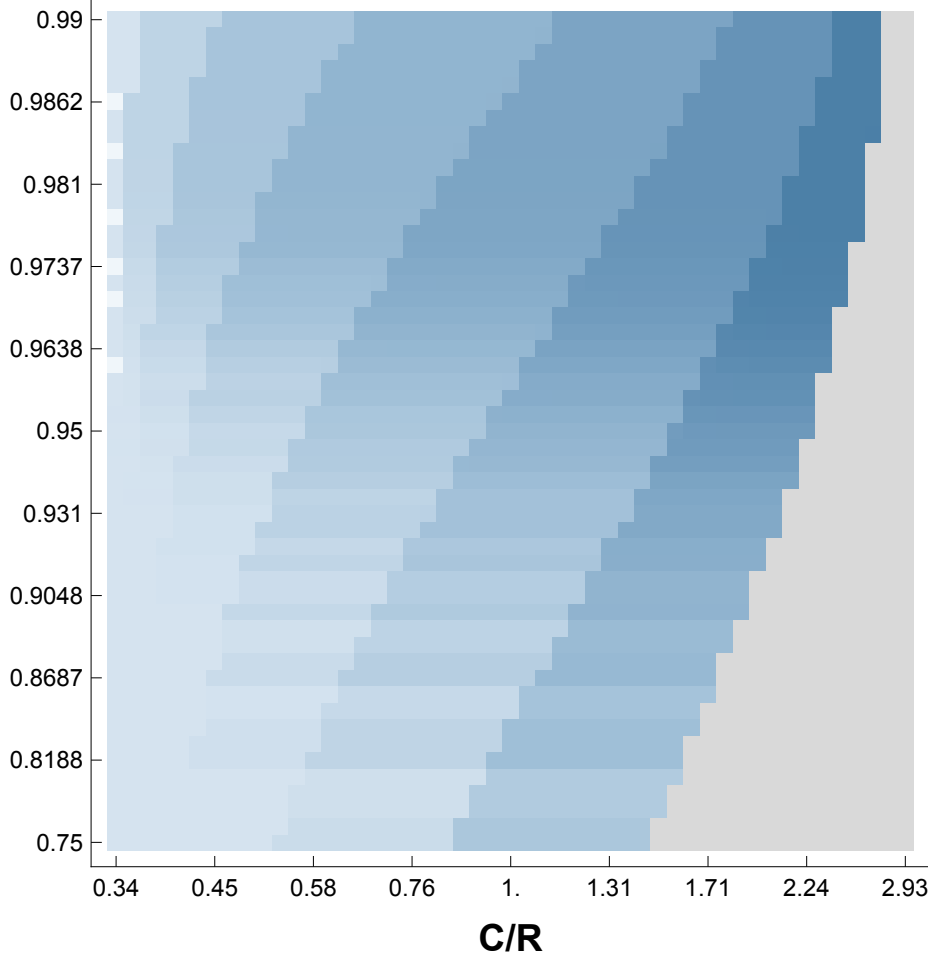

Supplement: Supplementary Data [file supp_eow024_suppl_data.zip › DCRatioFigureLabeled.pdf]

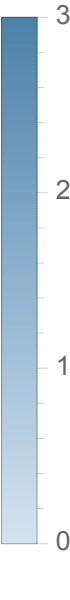

Supplement: Supplementary Data [file supp_eow024_suppl_data.zip › DCRatioLegend.pdf]

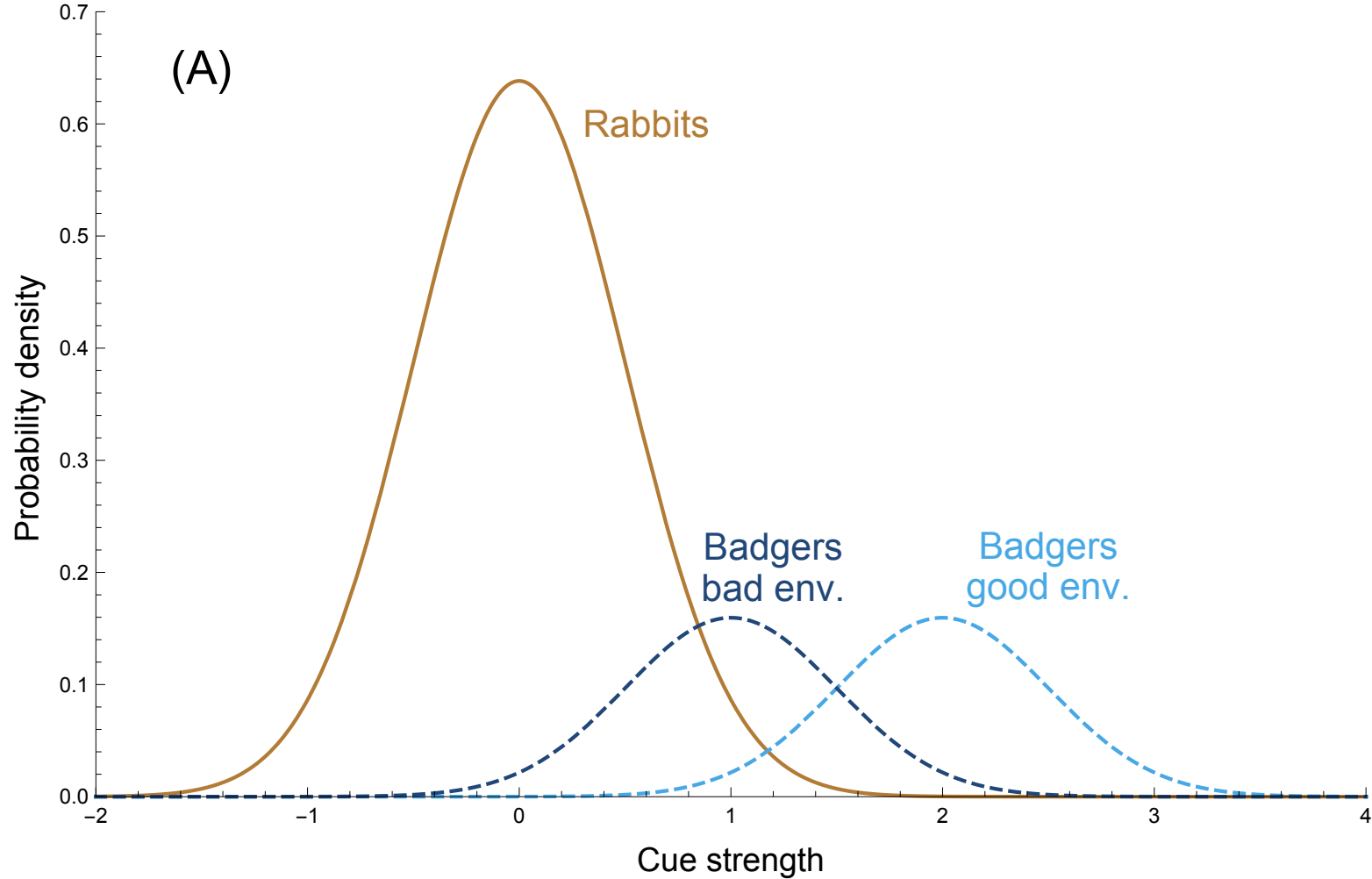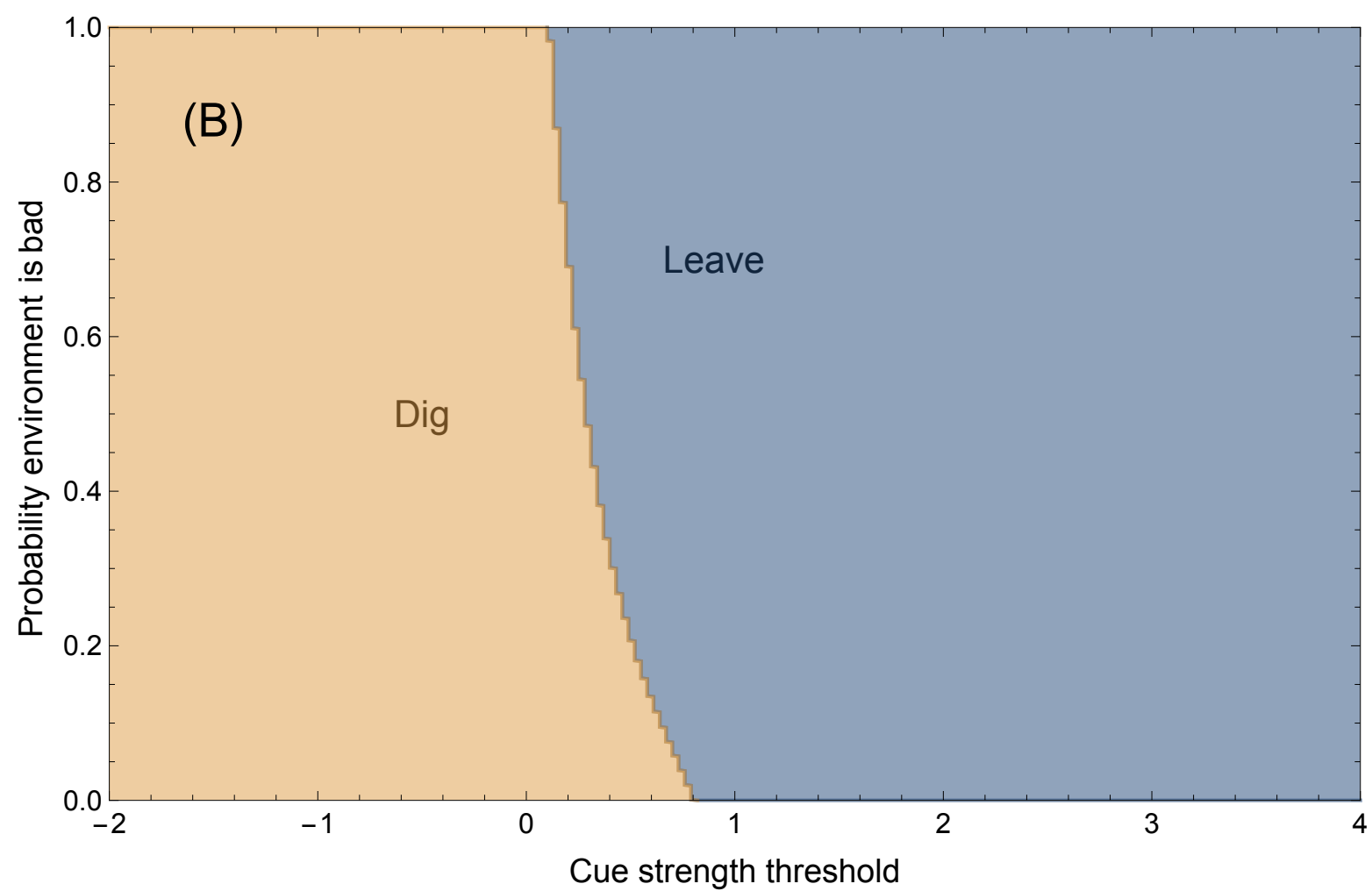

Supplement: Supplementary Data [file supp_eow024_suppl_data.zip › figure3_ctb.pdf]

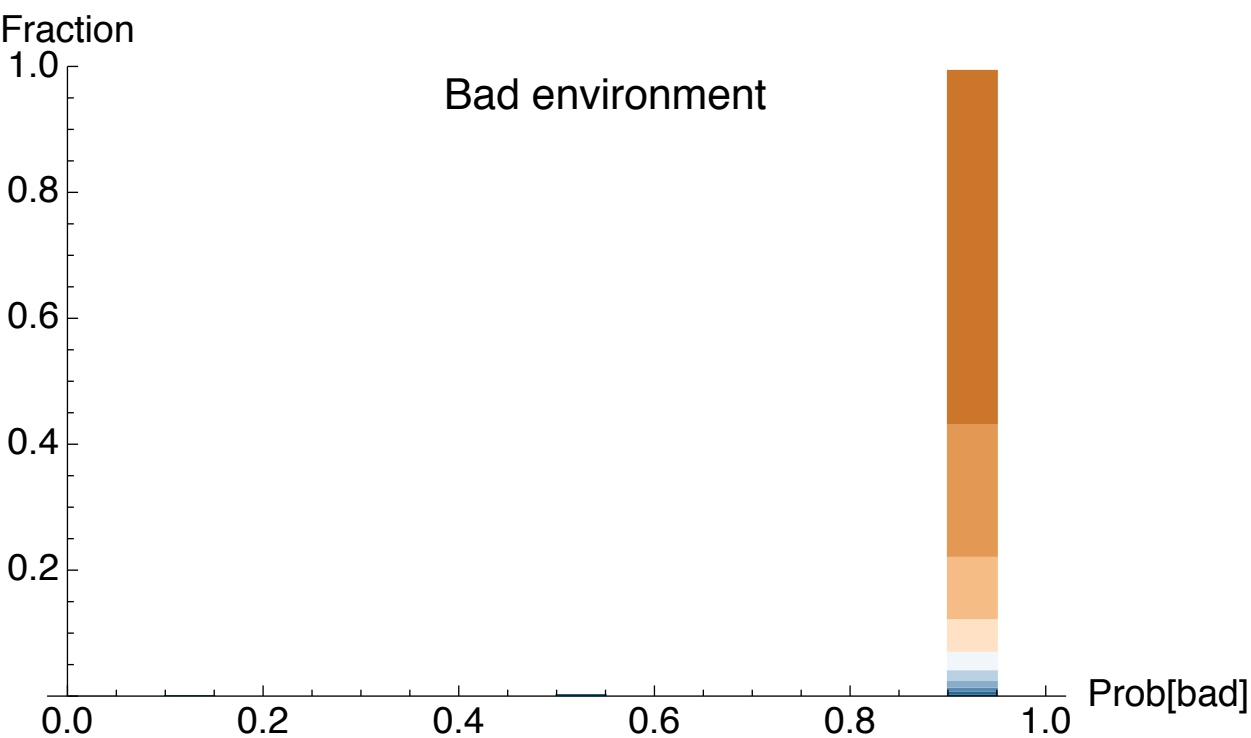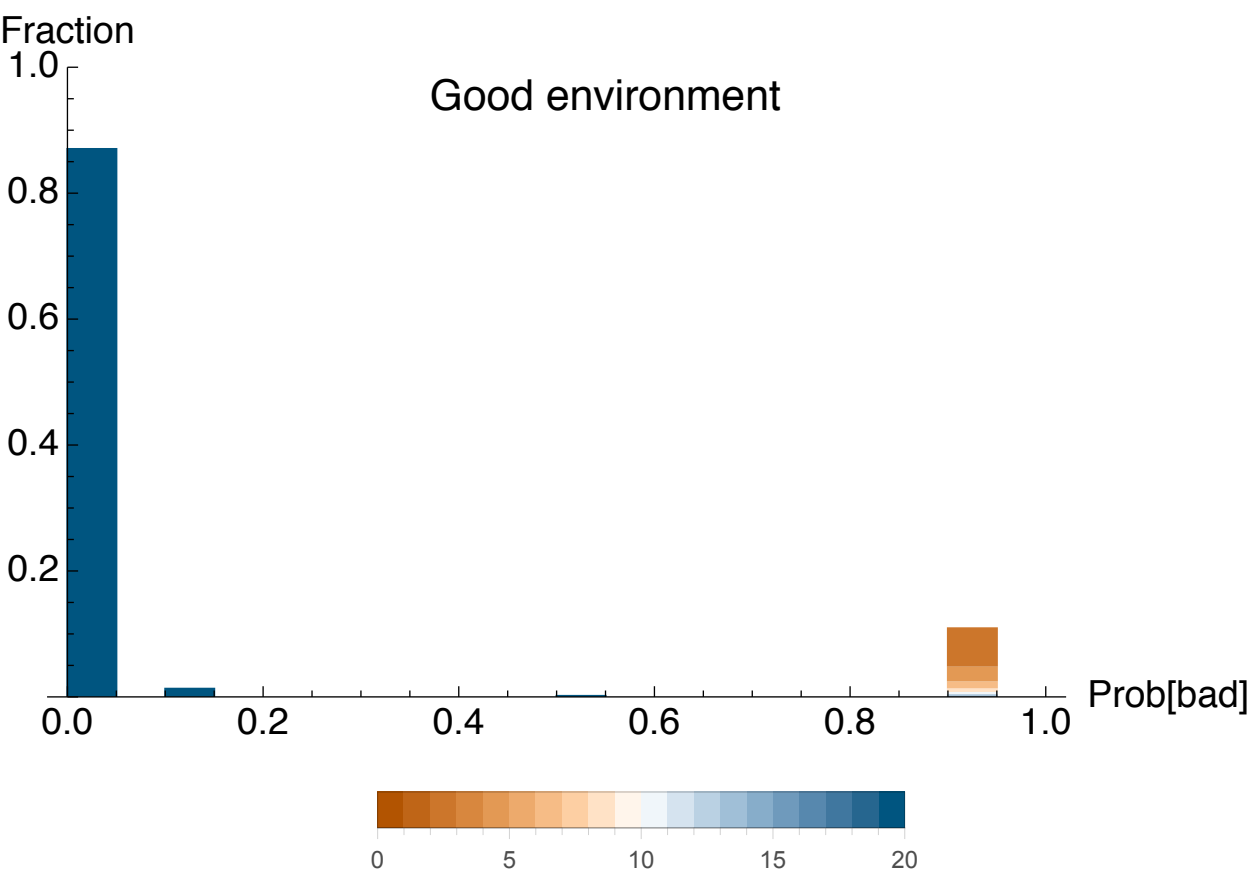

Supplement: Supplementary Data [file supp_eow024_suppl_data.zip › FigureModel1.pdf]

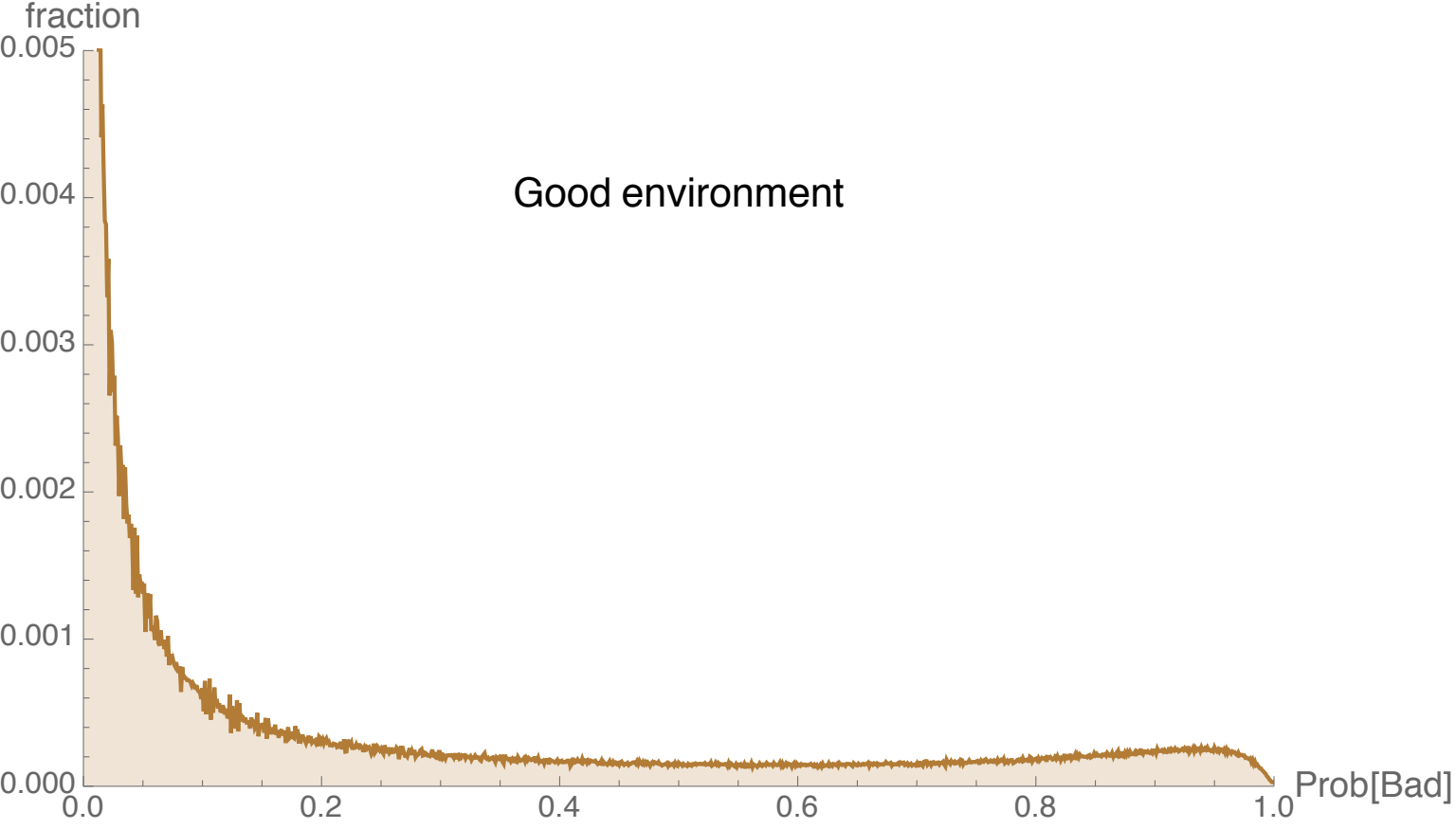

Supplement: Supplementary Data [file supp_eow024_suppl_data.zip › GoodEnvironment.pdf]

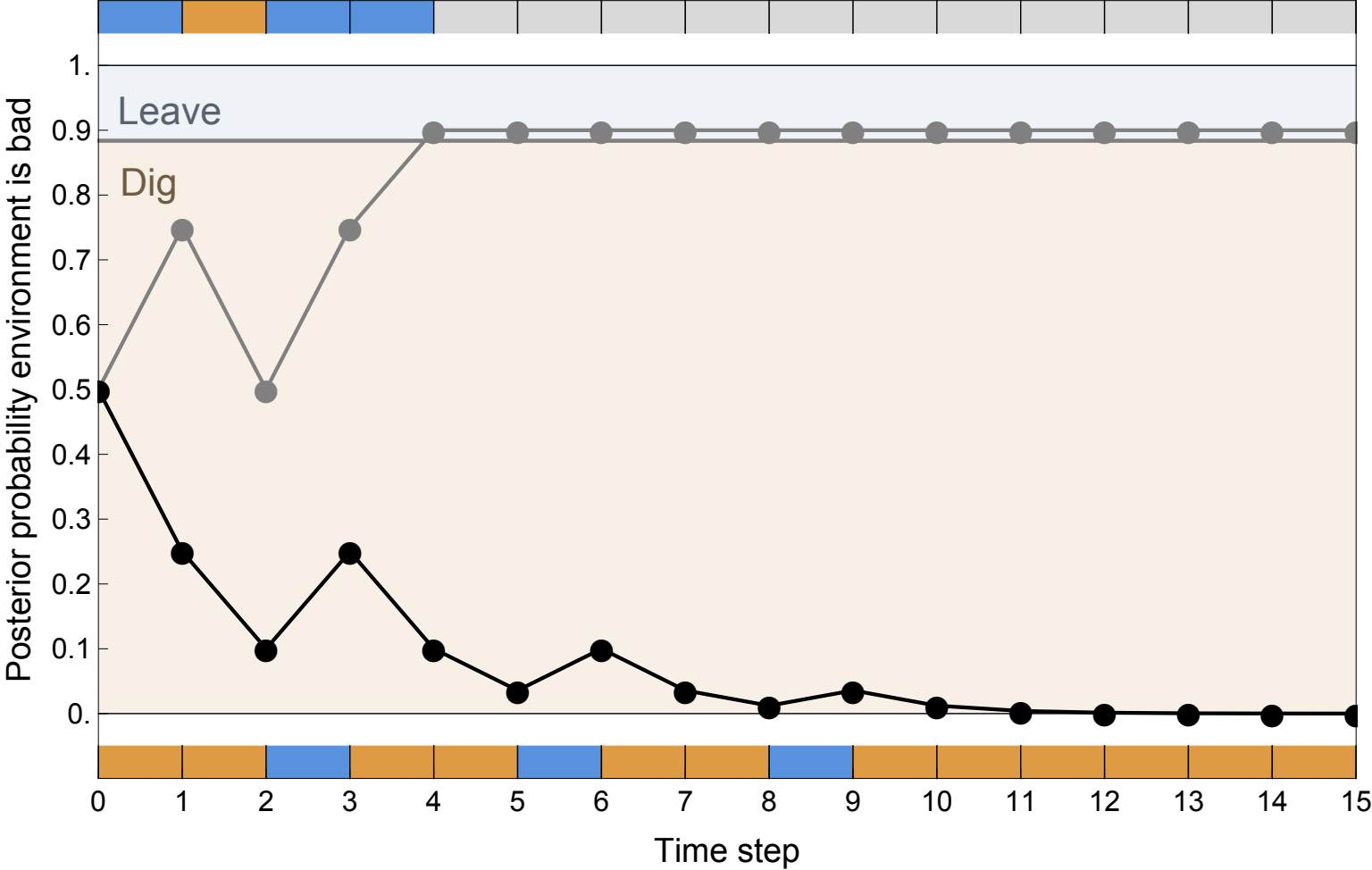

Supplement: Supplementary Data [file supp_eow024_suppl_data.zip › NewFig1.pdf]

Bad World

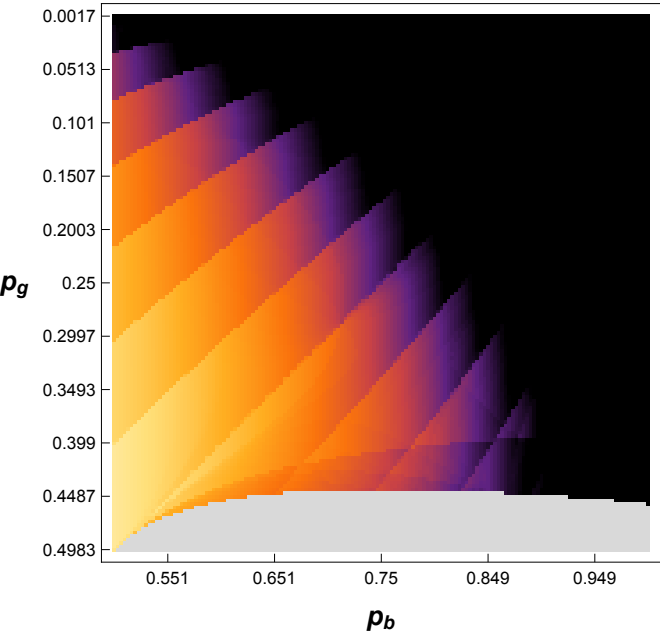

Good World

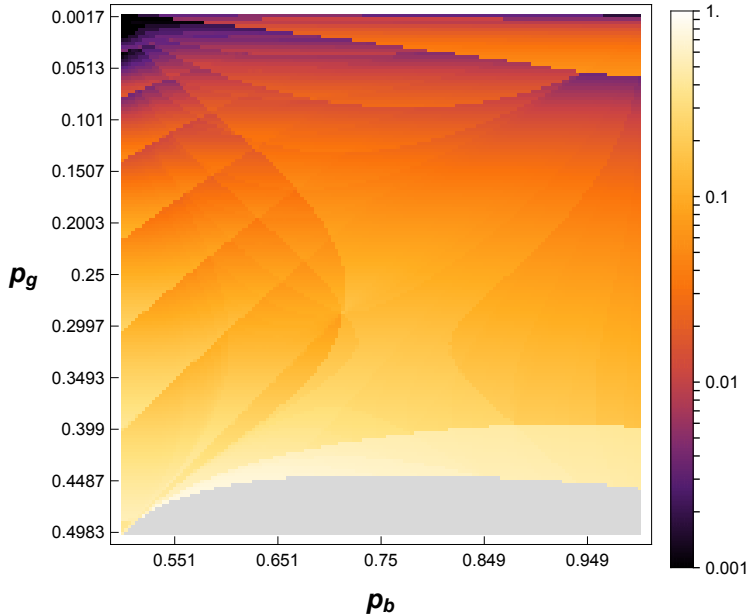

Supplement: Supplementary Data [file supp_eow024_suppl_data.zip › PgPbBadGoodFigureLabeled.pdf]

Log ratio of fraction mistaken in Good:Bad

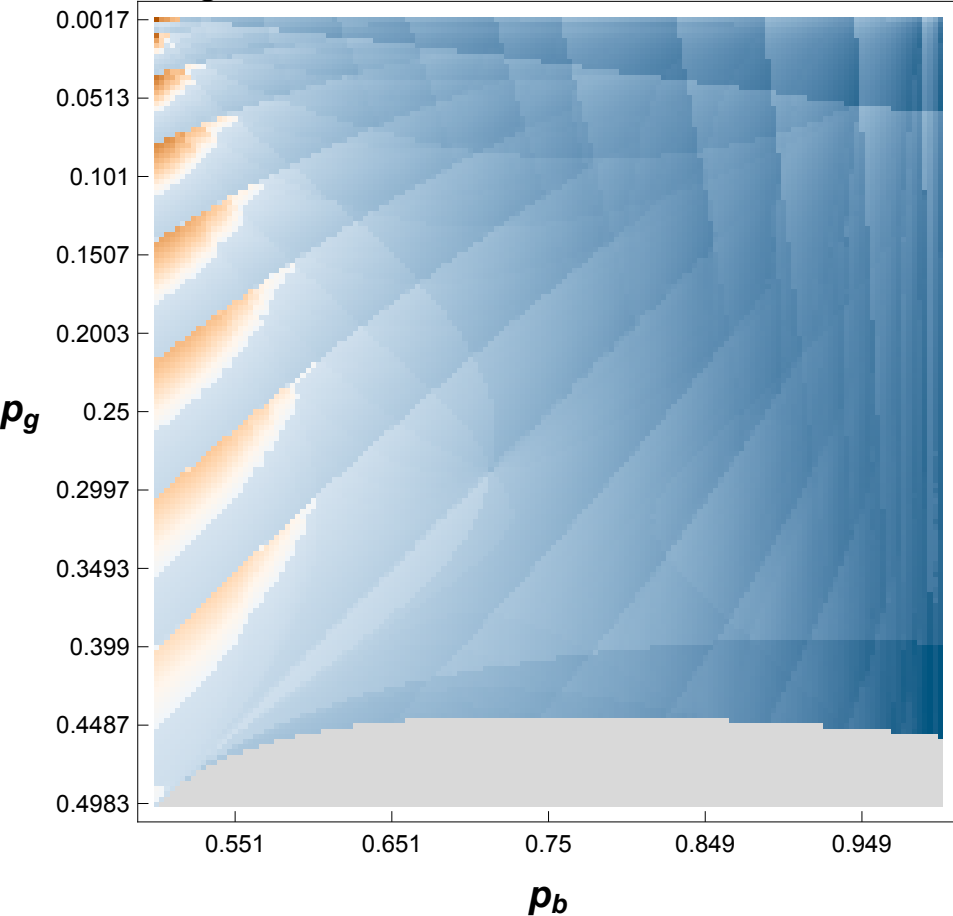

Supplement: Supplementary Data [file supp_eow024_suppl_data.zip › SensitivityToPgPbRatioGraphic.pdf]
